# Understanding local immunity to enable regionalized medicine

**DOI:** 10.1038/s44318-024-00255-6

**Published:** 2024-10-09

**Authors:** Marco De Giovanni, Donato Inverso, Matteo Iannacone

**Affiliations:** 1 Division of Immunology, Transplantation, and Infectious Diseases, IRCCS San Raffaele Scientific Institute, Milan, Italy; 2 Vita-Salute San Raffaele University, Milan, Italy; 3 Experimental Imaging Center, IRCCS San Raffaele Scientific Institute, Milan, Italy

**Keywords:** Immunology, Molecular Biology of Disease

## Abstract

This commentary of the *Sparks of Science* series from the Catalysts program discusses how dissection of local immunity paves the way for the selective targeting of specific niches in future therapeutics.

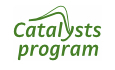

## Teaser

The adaptation of immune cells to their local microenvironments in our body is central to tissue homeostasis and linked to disease. In this commentary, we envision how future therapeutics could leverage local immunity to selectively target specific niches for disease treatment.

**Figure 1:**
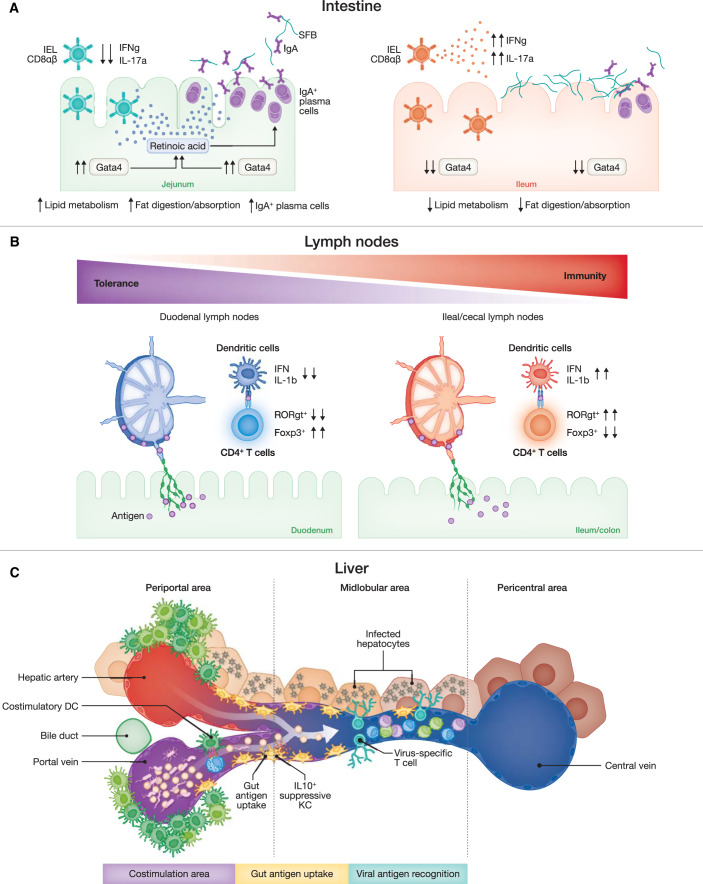
Regionalized Immunity in the Intestine, Lymph Nodes, and Liver. This figure illustrates the regionalized immune responses within the intestine, gut-associated lymph nodes (gLNs), and liver, highlighting the spatial organization and function of immune cells in these tissues. The figure highlights the importance of spatially restricted immune responses in maintaining tissue-specific immunity and suggests potential therapeutic interventions that target specific immune niches to modulate local immune responses effectively. (**A**) Intestine: The transcription factor GATA4 is crucial in regulating local immunity in the proximal small intestine. In the jejunum (left panel), GATA4 expression supports the maintenance of high luminal IgA levels by influencing retinol metabolism, which in turn limits the colonization of segmented filamentous bacteria (SFB). In addition, GATA4 expression promotes a tolerant population of intraepithelial lymphocytes (IELs), contributing to local immune tolerance. In contrast, in the ileum (right panel), the absence of GATA4 is associated with increased levels of pro-inflammatory IELs, which produce IFNγ and IL-17, decreased IgA-secreting plasma cells, and elevated SFB colonization, suggesting a more inflammatory environment. (**B**) Lymph Nodes: gLNs display regional specificity corresponding to the gut segment they drain. The figure shows that dendritic cells (DCs) and T-cell responses are differentially polarized depending on the gLN location. In duodenal gLNs (left panel), tolerogenic DCs (characterized by the lack of IFN and IL-1β expression) promote the differentiation of Foxp3+ regulatory T cells, supporting immune tolerance. Conversely, in the gLNs draining the distal gut (right panel), inflammatory DCs drive the differentiation of RORγt+ pro-inflammatory T cells, indicating a predisposition toward immune activation in response to the same luminal antigens. (**C**) Liver: Immune functions in the liver are spatially organized along the lobular axis, from the periportal to the central venous areas. The periportal region (left) is enriched with gut-derived metabolites and bacterial products, creating a pro-inflammatory environment. Here, Kupffer cells (KCs) produce immunosuppressive molecules such as IL-10, limiting inflammation and protecting against systemic bacterial spread. This area is also enriched with dendritic cells, forming an immune co-stimulation niche. In the mid-lobular area (middle), unique endothelial features support antigen recognition on hepatocytes by intravascular T cells. Exogenous IL-2 treatment can enhance the co-stimulatory potential of mid-lobular Kupffer cells, extending the co-stimulation area and restoring the function of virus-specific immune cells. The pericentral region (right) is less populated with immune cells, indicating a reduced immune surveillance environment. Parts of the figure were created with BioRender.com.

Much like the diverse countries and regions on a geographical map, our body is divided into large functional units, the organs, which are further divided into specialized microanatomical regions (or niches), each with distinct biochemical properties. Like a seasoned explorer visiting different regions around the globe, immune cells travel within and between various niches, quickly adapting to local microenvironments (Hu and Pasare, 2013; Agace and McCoy, 2017; Casas et al, 2024). This adaptation leads to the development of region-specific immune responses within and across different organs, a phenomenon we will refer to as “regional immunity”. Regionalized immune responses are crucial for tissue homeostasis and are intricately linked to the pathogenesis of major diseases (Agace and McCoy, 2017; Casas et al, 2024). Here, we summarize recent findings highlighting the importance of regional immunity using the intestinal mucosa, lymph nodes, and liver as paradigmatic examples (Fig. 1). We use these examples to propose an innovative medical model for future treatments that we name “regionalized medicine”, which focuses on developing vaccination and other therapeutic interventions tailored to selectively modulate immune responses within specific tissues and niches.

## Regionalized immunity in the intestine

The intestine is a long, tube-like structure that extends from the stomach to the anus. Its primary functions include digesting food, absorbing nutrients and water, and eliminating waste. The intestine is not homogeneous; rather, it consists of several anatomically and functionally specialized segments, each exposed to distinct environmental influences. Different intestinal segments have evolved unique defense strategies, including regional immune-system adaptations (Agace and McCoy, 2017). Continuous communication between environmental factors and the immune system is critical for preserving this equilibrium. Disruptions in these signals, such as dysbiosis, can reshape immune cell composition and functionality, leading to chronic inflammation and a higher risk of infections (Agace and McCoy, 2017). Consistent with this perspective, a recent study demonstrated how the transcription factor GATA4 regionally regulates bacterial colonization and tissue immunity in the proximal small intestine by modulating retinol metabolism and luminal IgA levels. This regulation is crucial, as mice lacking GATA4 expression in the jejunum exhibit increased susceptibility to *Citrobacter rodentium* infection, and celiac patients with low GATA4 expression display metabolic alterations and increased IL-17 immunity (Earley et al, 2023).

Likewise, independent intestinal niches can play distinct immunological roles. The intestinal mucosa consists of a single layer of columnar epithelial cells and an underlying lamina propria, a layer of loose connective tissue and interstitial matrix that contains immune cells. Recent findings have uncovered a spatial mechanism of tolerance within the lamina propria, which actively regulates effector T-regulatory cell generation and maintenance to preserve intestinal homeostasis (Gu et al, 2024). This mechanism can be disrupted during inflammation, leading to the migration of CD103^+^SIRPα^+^ dendritic cells from lymphoid aggregates to the lamina propria (Gu et al, 2024). Similarly, localization within distinct intestinal niches can lead to divergent immunological properties, as highlighted by a recent study showing the functional diversification of dendritic cells depending on their location within the lamina propria or intraepithelial compartment in the gut (Rivera et al, 2022). Indeed, mature-like pro-inflammatory conventional dendritic cells type 2 (cDC2) from the lamina propria transmigrate to the intestinal epithelium in response to food-derived retinoic acid, where they are imprinted by environmental cues to acquire an immature-like phenotype and tolerogenic properties (Rivera et al, 2022). Collectively, these observations underscore the importance of regional immunity in preserving intestinal homeostasis in health and disease, opening exciting therapeutic possibilities for improved clinical treatments (Fig. 1A).

## Regionalized immunity in the lymph nodes

Lymph nodes are secondary lymphoid organs that play a crucial role in priming and regulating adaptive immune responses (Andrian and Mempel, 2003). They are distributed throughout the body and connected via the lymphatic system, receiving a constant flow of lymph from surrounding tissues (Andrian and Mempel, 2003). This lymph carries various substances, including metabolic byproducts, proteins, carbohydrates, lipids, pathogens, and immune cells, which collectively influence the nature and function of each lymph node (Andrian and Mempel, 2003). Accordingly, distinct lymph nodes exhibit functional variability, differing in their ability to generate immune responses (Casas et al, 2024). For instance, gut-associated lymph nodes exhibit immunological specificity related to the segment of the gut they drain (Esterházy et al, 2019). Stromal and dendritic cell gene signatures, along with the polarization of T cells against the same luminal antigen, differ between gut-associated lymph nodes. Specifically, gut-associated lymph nodes draining the proximal small intestine predominantly induce tolerogenic responses, while those draining the distal gut favor pro-inflammatory T-cell responses (Esterházy et al, 2019).

Intriguingly, lymph node co-drainage of different organs has been suggested as a mechanism of immune crosstalk between anatomically connected tissues. For instance, lymph nodes shared between the pancreas, intestine and liver regulate pancreatic homeostasis (Brown et al, 2023). Hepatic and pancreatic migratory dendritic cells (migDCs) display a stronger pro-inflammatory profile than those originating from the duodenum. Consequently, the integration of different types of migDCs in each shared lymph node affects the properties of pancreatic β-cell-reactive T cells, with increased gut-homing and tolerogenic phenotype in proportion to duodenal co-drainage. However, duodenal viral infections render non-intestinal migDCs and β-cell-reactive T cells more pro-inflammatory in all shared lymph nodes, leading to increased pancreatic islet lymphocyte infiltration (Brown et al, 2023). Altogether, these findings highlight the crucial role of distinct lymph nodes in regulating local immunity and immune crosstalk across connected tissues, emphasizing how specific lymph node targeting may enhance the efficacy of future vaccination and therapeutic strategies (Fig. 1B).

## Regionalized immunity in the liver

The liver is organized into hexagonally-shaped functional units called lobules, which are anatomically and functionally defined by a unique microvasculature system that connects the intestinal portal vasculature with the general circulation (Ficht and Iannacone, 2020; Kawashima et al, 2024). Blood is collected from the gut by the hepatic portal vein, which, along with the hepatic artery, generates a dual blood supply (Ficht and Iannacone, 2020; Kawashima et al, 2024). Arterial and venous blood mix in the liver capillaries (sinusoids), extending from the portal area to the centrilobular vein (Ficht and Iannacone, 2020; Kawashima et al, 2024). This flow creates a spatial gradient of oxygen, blood pressure, and metabolites (Ben-Moshe and Itzkovitz, 2019). Intriguingly, spatially resolved transcriptomics have revealed that about 50% of the hepatocyte transcriptome is differentially regulated along the liver lobule axis, from the portal area to the central vein (Halpern et al, 2017). This pattern of spatial gene regulation, termed “liver lobular zonation”, is primarily orchestrated by liver vascular endothelial cells (Inverso et al, 2021; Halpern et al, 2018). Recent research has identified various peculiarities in intra-hepatic immune responses correlated with the lobular organization of the liver, extending the concept of lobular zonation to the immune compartment.

Liver-resident immune cells are enriched in the periportal area, with different subsets exhibiting distinct zonation patterns (Guilliams et al, 2022). Dendritic cells, for example, are notably enriched in the periportal region, while Kupffer cells display a gradient that diminishes along the sinusoid (Guilliams et al, 2022). Natural killer (NK) cells are predominantly found in the mid-lobular area (Guilliams et al, 2022). The continuous exposure of the periportal region to high concentrations of metabolites and bacterial products from the portal circulation results in sustained MYD88-dependent signaling in endothelial cells (Gola et al, 2021). This mode of signaling creates a chemokine gradient centered around the periportal area (Gola et al, 2021), with CXCL9 produced by endothelial cells inducing a periportal enrichment of CXCR3-expressing Kupffer cells and natural killer T cells (Gola et al, 2021). The periportal niche is adept at preventing systemic bacterial dissemination (Gola et al, 2021) and limiting excessive inflammation at the liver’s entry point (Miyamoto et al, 2024). The latter function is due to the periportal enrichment of Kupffer cells characterized by high levels of immune-suppressive molecules such as IL-10 and MARCO, distinguishing them from Kupffer cells in other liver regions (Miyamoto et al, 2024). This pattern of regionalization creates a specialized periportal immune niche that contains bacterial spread and inflammatory reactions, preventing sepsis and protecting the centrilobular areas from acute inflammation and cell death.

Conversely, circulating leukocytes preferentially home to the mid-lobular area. Various leukocyte subsets, including granulocytes (McDonald et al, 2008), NK cells (Geissmann et al, 2005), and CD8^+^ T cells (Guidotti et al, 2015), are recruited within the liver capillaries rather than the post-capillary venules. This atypical homing pattern, crucial for immune surveillance of hepatotropic viral infections by intravascular CD8^+^ T cells, is facilitated by the unique features of Lyve-1^+^ vascular endothelial cells. Liver sinusoidal endothelial cells (LSECs) occupy a unique position between the vascular lumen (sinusoid) and a lymphatic-like space (i.e., the space of Disse). This arrangement aligns with the atypical expression of lymphatic endothelial cell identity markers, such as Vegfr3 and Lyve-1, which are present in the sinusoidal area but absent from neighboring large vessels. In contrast, typical vascular pathways, including those involving cell adhesion molecules, focal adhesion, and leukocyte trans-endothelial migration, are less active in the sinusoidal areas compared to those in large vessels located at the periphery (portal area) and the center (central vein) of the liver lobule (Inverso et al, 2021). The final immunological outcome is the generation of a unique docking site for circulating immune cells. Indeed, the unusual expression of Lyve-1 leads to an accumulation of hyaluronan within the sinusoidal lumen. This, combined with the reduced expression of other adhesion molecules, facilitates a CD44-hyaluronan interaction-dependent recruitment of circulating cells in the mid-lobular area (Guidotti et al, 2015).

In summary, the hepatic lobule has evolved a sophisticated system of parenchymal immune compartmentalization (Fig. 1C). Circulating immune cells preferentially home to the mid-lobular area, where they inhabit a specific niche characterized by a unique vascular phenotype, facilitating antigen recognition by CD8^+^ T cells. These cells are pivotal in mounting an antigen-specific immune response to hepatotropic viral infections. Conversely, tissue-resident immune cells are concentrated in the periportal area, where they play a crucial role in the immunosurveillance of gut-derived pathogens, preventing systemic inflammation.

This immune compartmentalization generates a “liver paradox” where CD8^+^ T-cell priming (signal 1) and co-stimulation (signal 2) are spatially segregated. The sinusoidal area is optimized for hepatocellular antigen recognition, whereas professional APCs are predominantly located in the periportal region. This aligns with observations indicating that, under certain experimental conditions, effector CD4^+^ T cells can license periportal type 1 conventional dendritic cells (cDC1s) to promote the optimal expansion of CD8^+^ T cells that were originally primed elsewhere (English et al, 2024). In this context, the discovery that exogenous IL-2 can reverse the dysfunctional T-cell priming occurring in the mid-lobular area by promoting Kupffer cell cross-presentation represents a nascent form of regionalized immune therapy (Simone et al, 2021). While DCs are confined to the portal region, Kupffer cells are spread from the portal area and extend more broadly throughout the liver (Guilliams et al, 2022). Consequently, IL-2 administration could act by expanding the co-stimulatory region, thereby resolving the liver paradox and improving immune responses.

## Regionalized immunity for regionalized medicine

While our understanding of regional immunity is still quite limited, especially for human tissues, recent findings are beginning to elucidate the mechanisms that govern immune cell adaptation and function within different niches. Given the importance of local immune regulation in health and disease, we believe that future therapies should be designed to leverage these localized mechanisms for more effective results. For instance, selective antigen targeting to tolerogenic districts such as proximal gut-associated lymph nodes and lamina propria may allow for effective tolerance induction to restrain autoimmune and allergic processes in patients. Conversely, targeting antigens to more pro-inflammatory districts, such as the distal gut-associated lymph nodes, may be leveraged to induce effector immune responses upon mucosal vaccination. Similarly, re-establishing the local expression of important factors (e.g., GATA4) required for the maintenance of regional immunity in the gut and in the liver may improve the treatment of inflammatory conditions, such as celiac disease.

We propose the term “regionalized medicine” to describe the development of vaccination and clinical strategies tailored to selectively modulate immune responses within specific tissue niches or to direct immune cells towards or away from specific tissue niches. This approach represents a shift towards precision medicine, focusing on the distinct characteristics of different tissue niches to optimize therapeutic efficacy. The concept of regionalized medicine relies on a deep understanding of the unique immunological and biochemical environments of different regions in our body. While extensive research is needed to develop suitable technical approaches to achieve this challenging goal, a few biotechnological strategies designed to maximize targeting of specific organs or niches are emerging. Indeed, selective lymph node targeting of vaccines or drugs can be achieved by bioengineering (Schudel et al, 2019; Liu et al, 2014). However, future research is required to develop the ability to target specific lymph nodes in our body. Similarly, environment-sensitive molecule carriers, such as hypoxia or pH-sensitive liposomes (Li et al, 2019; Hasan et al, 2023), may boost the targeting of niches with defined biochemical properties, but future work is necessary to optimize this approach for selective niche targeting. In addition, recent tools such as vaccine-conjugated bi-specific antibodies (Labrijn et al, 2019), a family of antibodies designed to recognize two different antigens, may be optimized to simultaneously target two distinct cell types known to interact in a defined region. Finally, lentiviral vectors (Annoni et al, 2019) may be leveraged to re-establish zonated expression of key genes in specific tissue niches and restore regional immunity in the context of inflammation.

With our understanding of the mechanisms regulating regional immunity expected to grow exponentially in the coming years, we propose leveraging this expanding knowledge by establishing the new medical model of regionalized medicine. This model aims to develop vaccination or therapeutic interventions that target specific niches within the body, thereby fine-tuning immune responses at a regional level to boost treatment efficacy and reduce systemic side effects.
